# Acute Cardiometabolic and Exercise Responses to Breakfast Omission Versus Breakfast Consumption in Adolescent Girls: A Randomised Crossover Trial

**DOI:** 10.3390/nu15143210

**Published:** 2023-07-19

**Authors:** Julia K. Zakrzewski-Fruer, Victoria Morari, Rachael B. Champion, Daniel P. Bailey, Louise E. Ferrandino, Rebecca L. Jones

**Affiliations:** 1 Institute for Sport and Physical Activity Research, School of Sport Science and Physical Activity, University of Bedfordshire, Bedford MK41 9EA, UK; viktoriamorari@gmail.com (V.M.); rachaeland6@gmail.com (R.B.C.); louise.ferrandino@beds.ac.uk (L.E.F.); rebjones@lincoln.ac.uk (R.L.J.); 2 Centre for Physical Activity in Health and Disease, Brunel University London, Kingston Lane, Uxbridge UB8 3PH, UK; daniel.bailey@brunel.ac.uk; 3 Division of Sport, Health and Exercise Sciences, Department of Life Sciences, Brunel University London, Uxbridge UB8 3PH, UK; 4 Health Advancement Research Team (HART), School of Sport and Exercise Science, University of Lincoln, Lincoln LN6 7TS, UK

**Keywords:** adolescents, children, exercise, health, physical activity, nutrition

## Abstract

Girls often begin to skip breakfast during adolescence. This study compared the acute effect of breakfast omission versus consumption on cardiometabolic risk markers and perceived appetite and mood during rest and/or exercise in adolescent girls classified as habitual breakfast consumers. Girls (aged 13.2 ± 0.7 years) completed two 5.5 h conditions in a randomised crossover design: breakfast omission (BO) and standardised breakfast consumption (BC). A standardised lunch was provided at 3 h. Incremental cycling exercise was performed at 5 h. Blood and expired gas samples were taken at regular intervals. Whilst pre-lunch plasma glucose, insulin, and Metabolic Load Index incremental area under the curve (IAUC) were significantly lower in BO versus BC, post-lunch differences were reversed and larger in magnitude. Peak plasma glucose and insulin were significantly higher in BO versus BC. Pre-lunch perceived fullness and hunger were significantly lower and higher, respectively, in BO versus BC. Perceived energy and concentration were lower, and tiredness was higher, in BO versus BC. Exercise peak fat oxidation and Fatmax were unaffected. The lower physical activity enjoyment in BO versus BC approached significance. To conclude, acutely omitting breakfast adversely affects cardiometabolic risk markers and exercise enjoyment among adolescent girls who habitually consume breakfast.

## 1. Introduction

Prevention is more effective than a cure, and moderating postprandial glycaemia and insulinaemia is integral to cardiometabolic disease prevention, including type 2 diabetes (T2D) and cardiovascular disease (CVD) [1]. Even among healthy populations, repeated postprandial glycaemic excursions typical of waking hours can cause oxidative stress, inflammation, and atherosclerosis, progressing to cardiometabolic disease over time [1]. The increased prevalence of insulin resistance and T2D among young generations is particularly alarming due to the consequent prolonged health and economic burden when compared with older generations [2,3,4]. Adolescence is a particularly vulnerable time of life in the context of exacerbated glycaemia and T2D risk [5] due to transient pubertal insulin resistance, which disproportionally affects girls [6]. This becomes even more problematic for these adolescent girls when coupled with poor dietary and physical activity habits [7,8]. Breakfast consumption frequency and physical activity levels decline in the child-adolescent transition and are lower in girls versus boys [9,10,11,12], with only ≈20% of UK adolescent girls consuming breakfast daily [9] and ≈9% being sufficiently active [13]. The interaction of breakfast and physical activity is of further relevance, as consuming breakfast may acutely promote higher physical activity among adolescents [9,14]. For these reasons, adolescent girls are an important target population for interventions involving breakfast and physical activity to improve glycaemic control, where targeting this young population has potential for lifelong impact [15].

In adults, omitting breakfast exaggerates glycaemic and insulinaemic responses to subsequent meals compared to when breakfast is consumed; this has been termed the ‘second-meal effect’ [16,17,18]. Such responses may partly explain the association of infrequent breakfast consumption with cardiometabolic risk markers in children and adolescents [19,20,21] and T2D and CVD manifestation in adults [22,23]. Yet, due to the unique hormonal and metabolic profiles of adolescent girls, findings from adults may not be relevant to this relatively neglected but important population. The ≈32% reduction in insulin sensitivity that occurs from pre- to mid-puberty [6] suggests that eating earlier in the day, when insulin sensitivity and glucose tolerance are highest, may be particularly important for adolescents [24,25]. Further, increased glucose conversion into muscle glycogen has been highlighted as a primary mechanism for the second-meal effect in adults [18]. However, adolescent boys have a reduced muscle glycogen storage capacity and rely more on exogenous glucose and fat as fuels, and less on endogenous glucose, compared with adults until mid-to-late puberty [26,27]. That said, such evidence among females is limited and has used a narrow range of pubertal stages [28]. The slower rate of gastric emptying that may further contribute to the second-meal effect in adults [29] also depends on puberty, age, and sex, with slower rates reported in girls than boys and during mid-to-late versus pre-puberty [30]. Yet, existing second-meal effect research in adolescents has pooled data from overweight/obese 13- to 20-year-olds with little concern for the possible influence of pubertal status and sex on glycaemic and insulinaemic responses [31,32]. These studies have also included acclimation periods, which does not allow for a true examination of any acute effects. Further, a focus on apparently healthy adolescents has greater relevance for preventative efforts. Given the close coupling of glucose and triacylglycerol (TAG) metabolism and additive impact on cardiometabolic disease [33], TAG responses also warrant investigation. The primary aim of this study was to compare the effect of breakfast omission versus breakfast consumption on cardiometabolic risk marker responses to lunch in adolescent girls classified as habitual breakfast consumers. Resting and exercise substrate oxidation, perceived appetite and mood, and exercise enjoyment were assessed as secondary outcomes. It was hypothesised that cardiometabolic risk markers would be adversely affected by breakfast omission versus breakfast consumption, primarily due to higher post-lunch glycaemia and insulinaemia, as would mood perceptions and exercise enjoyment.

## 2. Materials and Methods

### 2.1. Participants and Recruitment

The current study was conducted in accordance with the University of Bedfordshire Research Ethics Committee’s ethical standards (number: 2018ISPAR018) and the Helsinki Declaration of 1975 as revised in 1983. The study was registered at clinicaltrials.gov (identifier NCT04476693). Data collection was completed between December 2018 and July 2019 in the Sport and Exercise Science Laboratories at the University of Bedfordshire. Girls were recruited from schools in Bedford, England. Parental informed consent and child assent were gained for all participants. Inclusion criteria were being female, being aged 12 to 14 years, attending a school in Bedford, and being classified as a habitual breakfast consumer. This age range is highly relevant as it typically coincides with the mid-pubertal drop in insulin sensitivity and the decline in breakfast consumption and physical activity levels among girls [6,7,9,10,11,12]. Habitual breakfast consumers were targeted on the basis that they may be more sensitive to the effects of breakfast manipulation than habitual breakfast skippers [34]. Individuals were excluded if they had relevant health-related issues (e.g., allergies to the test meals), were unable to exercise on a cycle ergometer, or were classified as a habitual breakfast skipper during preliminary measures. We did not specifically target overweight/obese or glucose-intolerant girls to ensure the findings were focused on disease prevention.

### 2.2. Sample Size Estimation 

**Figure 1:**
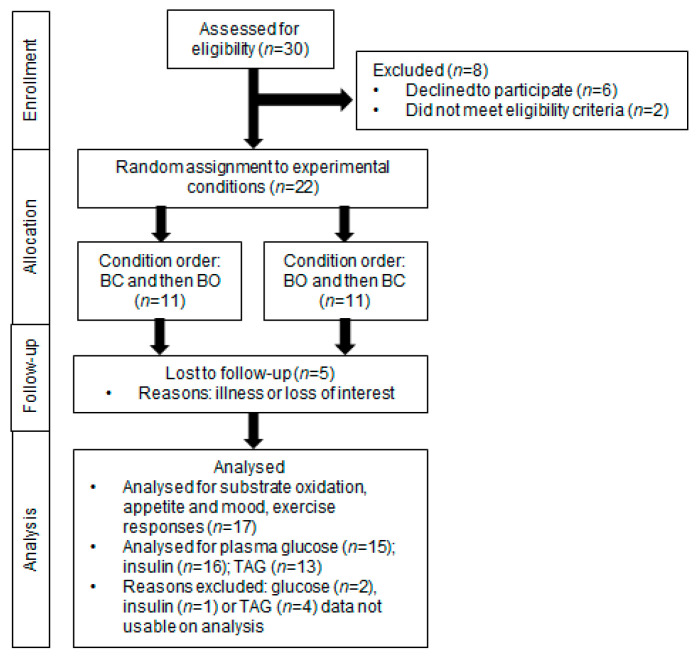
Participant flow chart of adolescent girls who participated in a randomised crossover trial comparing breakfast omission (BO) with breakfast consumption (BC).

A sample size of 16 adolescent girls was estimated to detect a significant difference in glucose incremental area under the curve (IAUC) at 80% power with an α of 0.05 and a Cohen’s d effect size of 0.72 [31] in this two-treatment crossover design. To allow for a ≈20% dropout rate, 22 participants were recruited (see Figure 1).

### 2.3. Preliminary Measurements

Stature was measured using a stadiometer (Holtain, Crymych, UK) to the nearest 0.01 m. Body mass was measured to the nearest 0.1 kg and percent body fat was estimated to the nearest 0.1% using a Tanita Body Composition Analyzer (BC-418 MA, Tanita Corporation, Tokyo, Japan). Body mass index (BMI) was calculated as body mass divided by stature squared (kg∙m^−2^). Waist circumference was measured to the nearest millimetre in line with recommended procedures [35]. Physical maturation was self-assessed by the participants with the assistance of a parent or primary caregiver using validated secondary sexual characteristics scales [36,37]. International age- and sex-specific BMI cut-offs were used to classify weight status [38]. To assess breakfast habits, a questionnaire was administered where participants were asked about consumption frequency (number of days per week), time and location of consumption, types of food and beverages consumed, and reasons for skipping breakfast for weekdays and weekend days. Only those who reported that they normally consumed at least 50 kcal before 10:30 on four to seven days per week were eligible to take part [16,39]. The girls were provided with the opportunity to ask questions about the breakfast habits questionnaire to clarify their understanding and were asked to confirm all of their responses before participating.

### 2.4. Experimental Design

Using a randomised cross-over design, each participant completed two, 5.5 h experimental conditions in a controlled laboratory setting: breakfast omission (BO) and breakfast consumption (BC). The conditions were completed in a random, counter-balanced order determined using a computer-based random number generator. VM and RBC generated the random allocation sequence, enrolled the participants, and assigned participants to the experimental conditions. With the assistance of a parent or primary caregiver, the girls were asked to record their weighted food and drink intake, refraining from caffeine, in the 48 h period before their first experimental condition using a weighed food diary and digital scales (Salter, 1036BKSSDR, HoMedics Group Ltd., Kent, UK). The girls were then asked to replicate this diet before their second experimental condition. Due to the potential for acute physical activity to enhance glucose control and insulin sensitivity among adolescents [40,41], the participants were also instructed to refrain from moderate-to-vigorous physical activity in the 48 h preceding both experimental conditions. Participants completed all sessions in friendship pairs to help maximise comfort and adherence. The conditions were conducted 7 to 30 days apart to avoid carryover effects and for logistical reasons. Due to the irregular menstrual cycle patterns of adolescent girls and in line with previous research [28,42], it was not possible to align experimental conditions to the early follicular phase when the adult literature suggests ovarian hormones are most stable and thus may impact metabolism minimally [43,44].

The experimental conditions started after an overnight fast and a 20 min rest at ≈08:15. First, an expired gas sample was collected for the estimation of resting metabolic rate (RMR) and substrate oxidation [45,46]. Immediately after, a capillary blood sample was collected for the measurement of fasting plasma glucose, insulin, and TAG, and subjective appetite and mood were assessed. These measures were repeated at regular intervals over the subsequent 5 h. Immediately after baseline measures, a standardised breakfast was consumed in BC or an amount of water equivalent to the fluid content of the standardised breakfast was consumed in BO. A standardised lunch was consumed at 3 h, which was followed by a 2 h post-lunch postprandial period and an incremental exercise protocol at 5 h. Participants remained sedentary until the incremental exercise protocol. They were permitted to spend their time carrying out activities to reduce boredom, such as reading, writing, and watching DVDs. Water intake was permitted ad libitum during the first experimental condition, with the quantity and timings replicated in the second experimental condition.

### 2.5. Test Meals

The characteristics of the standardised breakfast, including quantity, composition, and timing, aligned with proposed definitions of ‘breakfast’ [47,48]. The breakfast consisted of All-Bran Original cereal (Kellogg’s, Trafford, UK), semi-skimmed milk (Tesco, Welwyn Garden City, UK), Royal Gala apple (Tesco, Welwyn Garden City, UK), strawberry flavoured yoghurt (Yeo Valley, Blagdon, UK), and orange juice (Tesco, Welwyn Garden City, UK). The percentage of energy from macronutrients was 70% carbohydrates, 15% fat, and 16% protein. The calculated glycaemic index (GI) of the breakfast was 42 based on the International Table of GI and Glycaemic Load values [49] and the weighted means of the GI values for the component foods [50]. This carbohydrate-based, low-GI breakfast was chosen as the repeated ingestion of glucose may act as a physiological mechanism for the ‘second meal effect’ [51], with low-GI breakfasts being more beneficial than high-GI breakfasts [52,53]. Further, high-fibre, low-GI, cereal-based breakfasts can reduce cardiometabolic disease risk among children and adolescents [19,54,55,56]. The breakfast was provided to the participants in quantities containing 0.06 g of carbohydrate per kcal of individual measured RMR based on the baseline expired air sample of the first experimental condition. This quantity has been shown to induce beneficial second meal responses in adults [16] and meant that the energy content aligned with recommendations that breakfast should contribute to ≈15–25% of daily energy intake [47]. The participants consumed the breakfast at ≈08:30, thus aligning with definitions that propose breakfast should be consumed within 2 to 3 h of waking and typically before 10:00 [47,48]. Our previous research indicated that this is an ecologically valid time of day to consume breakfast among adolescent girls [14,39].

The standardised lunch meal was composed of white bread without crust (Tesco, UK), margarine (‘Buttery Spread’, Tesco, UK), strawberry jam (Tesco, UK), salted crisps (Walkers, Reading, UK), and sparkling glucose drink (Lucozade Energy Original, Coleford, UK). The percentage of energy from macronutrients was 67% carbohydrate, 27% fat, and 6% protein. The calculated GI was 75 [49,50]. This carbohydrate-rich, high-GI lunch was selected on the basis that it would challenge glycaemic control among adolescent girls [57] and reflects previous work in adults [16,17,18]. The lunch was provided to the participants in quantities containing 0.08 g of carbohydrate per kcal of measured RMR. This ensured that it was larger than the breakfast meal and was an ecologically valid portion. Participants were asked to consume the breakfast and lunch meals within 15 min; consumption time during the first experimental condition was recorded and replicated during the second condition. Participants were required to consume the entire breakfast and lunch meal.

### 2.6. Blood Sampling and Analysis

Capillary blood samples (≈500 µL) were taken from a pre-warmed hand (i.e., 5 min in warm water) by finger prick (Haemolance+ Safety Lancet, Prospect Diagnostics, Dronfield, UK) into collection tubes (Microvette CB300 EDTA, Sarstedt Ltd., Leicester, UK) at 0 (baseline), 0.5, 1, 2, and 3 h pre-lunch and at 0.25, 0.5, 1, 1.5, and 2 h post-lunch. The samples were centrifuged for 5 min at 2000× *g* (Hawksley Haematospin 1300, Hawksley Medical and Laboratory Equipment, Lancing, UK). The plasma (≈250 µL) was subsequently removed and stored at −80 °C for future batch analysis. Plasma glucose and TAG concentrations were determined in duplicate using enzymatic, colorimetric methods (Randox, Crumlin, Northern Ireland, UK). Plasma insulin concentration was measured in duplicate using an enzyme-linked immunosorbent assay (Mercodia, Uppsala, Sweden). Total area under the curve (TAUC) and IAUC for the 3 h pre-lunch period and 2 h post-lunch period were calculated using the trapezium rule [50]. The intra-assay coefficient of variation of the duplicate samples was 3.3% for plasma glucose, 6.7% for plasma insulin, and 14.9% for plasma TAGl. The homeostatic model assessment of insulin resistance (HOMA-IR) was calculated as the product of the fasting glucose and insulin (averaged from BO and BC) divided by the constant 22.5 [58]. Age-, sex-, and BMI-specific percentiles of HOMA-IR were used to identify participants classified as ‘at risk’ of cardiometabolic disease [59]. Impaired fasting plasma glucose was determined according to the American Diabetes Association criteria (i.e., 5.6 to 6.9 mmol·L^−1^) [60]. The sum of glucose and TAG concentrations at each time point was taken as the Metabolic Load Index (MLI) [33].

### 2.7. Expired Gas Sampling and Indirect Calorimetry

Expired gas was sampled during rest for 10 min periods after the participant had lay supine on a bed for at least 20 min, in line with recommended procedures [45,46]. Resting samples were collected at 0 (baseline), 0.5, 1, 2, and 3 h pre-lunch and at 0.5, 1, 1.5, and 2 h post-lunch. Samples were also collected continuously throughout the incremental exercise protocol. A flow meter was attached to an appropriately sized facemask to collect breath-by-breath data using an online gas analysis system (Metalyzer 3B; Cortex, Leipzig, Germany). The data were interpolated into 5 s epochs. Oxygen consumption and carbon dioxide production data were then checked for a steady state with respiratory exchange ratio values exceeding 1.00 being removed in line with the assumptions of indirect calorimetry [45,61]. For each 10 min resting expired air sample, the first three minutes were excluded, and data corresponding to the lowest energy expenditure from a 5 min rolling average were used to reflect minimum individual energy needs. During exercise, data from the final minute of each stage were used. Substrate oxidation rates and total energy expenditure were estimated using stoichiometric equations, with the assumption that the urinary nitrogen excretion rate was negligible [61].

### 2.8. Incremental Exercise Protocol and Fatmax Estimation

The Fatmax incremental exercise protocol was completed 2 h after lunch to ensure that it did not interfere with the second-meal response and to reflect the ‘after school’ period when adolescents have opportunities to be physically active without school timetable restrictions [62]. The protocol consisted of seven, submaximal 4 min stages on a cycle ergometer (Lode, Groningen, The Netherlands). Incremental exercise tests with 3 min stages have been validated for Fatmax estimations in children [63]. A degree of caution was employed with slightly longer stages to increase the likelihood of a steady state being attained, as used previously in adolescents varying in weight status [41,64]. The cycle ergometer was set to 0 W for the first stage and then 20%, 30%, 40%, 50%, 60%, and 70% of individual theoretical maximal aerobic power (i.e., 3 W*kg fat free mass [65]), similar to previous work in adolescents [64,66]. Participants cycled at 60 rev∙min^−1^ throughout. Perceived exertion was assessed using the OMNI scale in the final 30 s of each stage. This scale contains both pictorial and verbal descriptors positioned along a numerical response range of 0 to 10, making it a valid, reliable, and preferred measure of perceived exertion among adolescent girls [67]. Exercise was terminated when the respiratory exchange ratio exceeded 1.00 (i.e., when substrate oxidation estimations via indirect calorimetry became invalid [61]). Expired gas was sampled, and heart rate was recorded throughout (Polar Vantage, Polar, Kempele, Finland). Fatmax (expressed as % maximum heart rate) and peak fat oxidation rate were estimated using individual best-fit polynomial curves of fat oxidation rate against % maximum heart rate using data from the final minute of each stage [41,42,63]. Exercise enjoyment was assessed 5 min after completion of the incremental exercise protocol using the 16-point Physical Activity Enjoyment Scale, as validated in adolescent girls [68].

### 2.9. Perceived Mood and Appetite

Perceptions of mood and appetite were assessed at baseline and every 30 min throughout the experimental conditions. Mood (energy, tiredness, tension, and calmness) was assessed using a 20-item modified version of the ‘Activation–Deactivation Check List’ short design specifically for, and used successfully, in adolescents [69]. Perceived hunger, fullness, and concentration were assessed using 100 mm visual analogue scales.

### 2.10. Statistical Analyses

Data were analysed using IBM SPSS statistics software for Windows version 26 (IBM Corporation, New York, NY, USA). Linear mixed models were employed to assess all outcome variables with condition and time as fixed factors. Plasma glucose, insulin, and TAG concentrations and resting substrate oxidation rates were examined as follows: (1) in their raw format over 10 and nine time points, respectively, and (2) as IAUC and TAUC over two time periods (pre-lunch (3 h) and post-lunch (2 h)). The linear mixed models included participants as a random effect and were adjusted for period (order) effects [70], with baseline values included as a covariate for resting cardiometabolic and substrate oxidation outcomes. Shapiro–Wilks tests and Q-Q plots were used to confirm the normality of the residuals. The main effect of condition is reported for all variables, whereas the main effect of time is reported only when the pattern differed between the conditions (i.e., when the condition by time interaction was significant). Significant condition by time interactions were followed up with post hoc analyses using the Holm–Bonferroni correction for multiple comparisons [71]. Statistical significance was accepted as *p* ≤ 0.05. To gauge the magnitude of potentially meaningful between-condition differences, Cohen’s d absolute standardised effect sizes (‘d’) are provided to supplement important findings, with 0.2 considered the minimum important difference, 0.5 moderate, and 0.8 large [72]. Values are presented as means with SDs for descriptive data, unless stated otherwise. For statistical analysis data, values are presented as estimated marginal means with 95% confidence intervals (CIs).

## 3. Results

### 3.1. Participants

The final sample included 17 participants. Figure 1 shows the flow of participants from enrolment to analyses. Table 1 shows the physical characteristics of the participants. Four participants were classified as overweight, and one was classified as obese [38]. None of the participants were classified as ‘at risk’ of cardiometabolic disease according to age-, sex-, and BMI-specific percentiles of HOMA-IR [59]. Three participants had an impaired fasting plasma glucose based on the American Diabetes Association criteria [60].

### 3.2. Test Meal Characteristics

Absolute energy and macronutrient intakes for the standardised breakfast and lunch are shown in Table 2.

### 3.3. Plasma Glucose, Insulin, and TAG Responses

**Figure 2:**
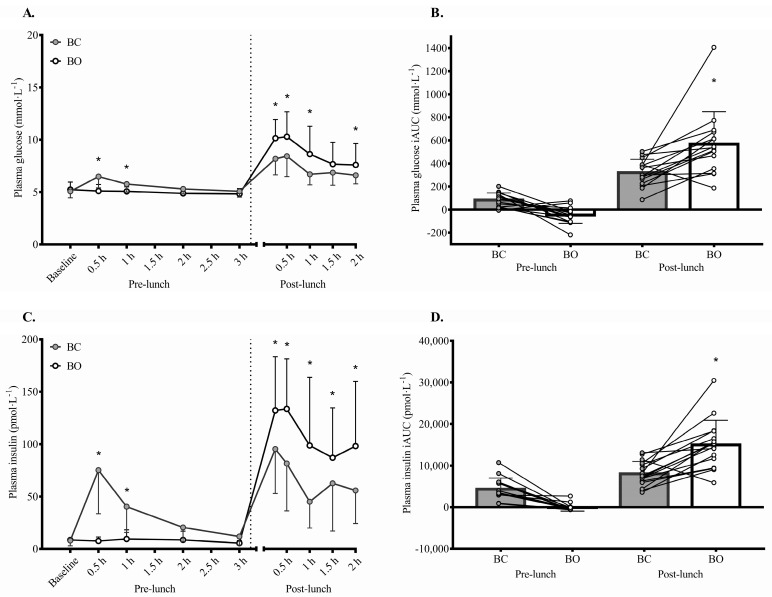
Plasma glucose (**A**), glucose incremental area under the curve (IAUC) (**B**), plasma insulin (**C**), and insulin IAUC (**D**) concentrations during the breakfast omission (BO) and breakfast consumption (BC) condition. Data are presented as means and SD. For panels (**B**,**D**), circles and lines represent individual data points. * denotes statistical significance (*p* < 0.05). BC was the consumption of a standardised breakfast providing 0.06 g of carbohydrate per kcal of measured resting metabolic rate. BO was the consumption of an amount of water equivalent to the fluid content of the standardised breakfast provided in BC. A standardised lunch providing 0.08 g of carbohydrate per kcal of measured resting metabolic rate was provided 3 h after BO or BC. The dotted lines denote the separation of the pre- and post- lunch periods. An incremental exercise protocol was performed 2 h after lunch. Statistical analyses were completed using linear mixed models with condition and time included as fixed factors.

**Figure 3:**
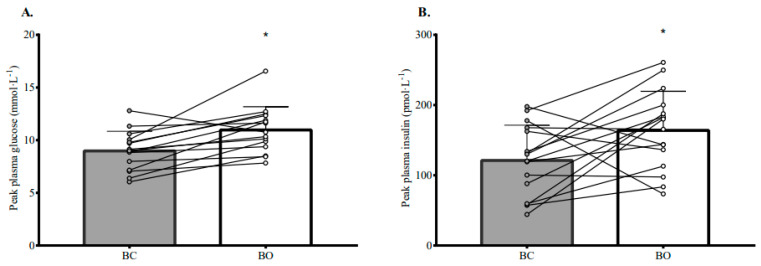
Peak plasma glucose (**A**) and peak plasma insulin (**B**) during the breakfast omission (BO) condition and breakfast consumption (BC) condition. Data are presented as means and SD. Circles and lines represent individual data points. * denotes statistical significance (*p* < 0.05). BC was the consumption of a standardised breakfast providing 0.06 g of carbohydrate per kcal of measured resting metabolic rate. BO was the consumption of an amount of water equivalent to the fluid content of the standardised breakfast provided in BC. A standardised lunch providing 0.08 g of carbohydrate per kcal of measured resting metabolic rate was provided 3 h after BO or BC. An incremental exercise protocol was performed 2 h after lunch. Statistical analyses were completed using linear mixed models. Grey bars and circles represent group and individual responses, respectively, to BC. White bars and circles represent group and individual responses, respectively, to BO.

Table 3 reports the AUC cardiometabolic risk factor results, and Figure 2 illustrates the results over time on a group and individual level for BO and BC. Plasma glucose concentration was significantly higher during BO versus BC with a moderate effect size (*p* = 0.004; d = 0.57). The significant condition by time interaction indicated that the condition effect depended on time (*p* < 0.0005), with plasma glucose concentration being lower in BO versus BC until lunch, after which this difference was reversed and became more pronounced (see Figure 2). Peak plasma glucose concentration was also higher in BO versus BC (*p* = 0.003; d = 0.93; see Figure 3). For plasma glucose IAUC and TAUC, the main effect of condition was not significant, whereas the main effect of time and condition by time interaction were significant (*p* < 0.0005). Pre-lunch glucose IAUC and TAUC were both significantly lower in BO versus BC with moderate to large effects (*p* ≤ 0.027; d = 0.75 to 0.83), whereas post-lunch glucose IAUC and TAUC were both significantly higher in BO versus BC with large effects (*p* ≤ 0.001; d = 1.15 to 1.47). 

Plasma insulin concentration was significantly higher in BO versus BC with a small, but almost moderate, effect size (*p* = 0001; d = 0.49). The significant condition by time interaction indicated that the condition effect depended on time (*p* < 0.0005), with plasma insulin concentration being lower in BO versus BC until lunch, after which a reversal and pronunciation of this difference was seen (see Figure 2). Peak plasma insulin concentration was higher in BO versus BC with a moderate effect size (*p* = 0.035; d = 0.78; see Figure 3). Although the main effect of condition was not significant for plasma insulin IAUC and TAUC, the main effect of time and condition by time interaction were significant (*p* < 0.0005). Pre-lunch insulin IAUC and TAUC were significantly lower in BO versus BC (*p* < 0.0005; d = 1.23 to 1.25), whereas post-lunch insulin IAUC and TAUC were significantly higher in BO versus BC (*p* ≤ 0.0005; d = 1.58 to 1.91); these effects were all large. 

Plasma TAG concentration was significantly lower in BO versus BC with a condition by time interaction (*p* ≤ 0.002) and a large effect size (d = 1.15) associated with the condition main effect. Main effects and the condition by time interaction were not significant for plasma TAG IAUC. There was a significant effect of condition for plasma TAG TAUC, reflecting lower concentrations during BO versus BC (*p* = 0.004; d = 0.95) with no condition by time interaction.

The main effect of condition was significant for MLI, which was higher in BO versus BC with a small effect size (*p* = 0.005; d = 0.32). This was accompanied by a significant condition by time interaction (*p* ≤ 0.0005), where MLI was lower pre-lunch at 0.5 to 2 h and higher post-lunch at 0.25 to 1 h and at 2 h in BO versus BC (*p* ≤ 0.038). Although the main effect for MLI IAUC and TAUC did not differ between conditions (*p* ≥ 0.264), there was a significant main effect for time and condition by time interaction (*p* ≤ 0.001). Pre-lunch, MLI IAUC was lower in BO versus BC (*p* = 0.034; d = 0.78); this effect was then reversed with a larger difference post-lunch (*p* = 0.001; d = 1.35). Similar results were found for MLI TAUC, which was lower in BO versus BC pre-lunch (*p* = 0.028; d = 0.74), with a reversal of this effect and larger difference post-lunch (*p* = 0.005; d = 0.97).

### 3.4. Resting Substrate Oxidation

The main effect of condition and the condition by time interaction were significant for resting fat and carbohydrate oxidation (*p* < 0.0005). The higher fat oxidation and lower carbohydrate oxidation in BO versus BC had large effect sizes (d = 1.65 and d = 2.18, respectively) and occurred at all time points other than baseline and the final time point. Total resting energy expenditure was lower in BO versus BC (*p* < 0.0005; d = 0.86). The condition by time interaction was also significant (*p* ≤ 0.009), with lower resting energy expenditure in BO versus BC from 15 min after breakfast to 15 min after lunch and at 1 h after lunch. 

Table 3 reports the AUC results for resting fat oxidation, carbohydrate oxidation, and energy expenditure for BO and BC. The main effect of condition and the condition by time interaction were significant for resting fat and carbohydrate oxidation IAUC and TAUC (*p* ≤ 0.016). Pre-lunch and post-lunch differences were all significant (*p* ≤ 0.041). Fat oxidation IAUC was higher pre-lunch (d = 2.40) and lower post-lunch (d = 1.03) in BO versus BC, with TAUC being higher pre-lunch (d = 2.04) and post-lunch (d = 0.93) in BO versus BC. In opposition, carbohydrate oxidation IAUC was lower pre-lunch (d = 3.14) and higher post-lunch (d = 0.71) in BO versus BC, with TAUC being lower pre-lunch (d = 3.10) and post-lunch (d = 1.44) in BO versus BC. The main effect of condition was significant for resting energy expenditure IAUC and TAUC (*p* ≤ 0.003), whereas the condition by time interaction was significant for IAUC only (*p* = 0.006). Resting energy expenditure IAUC was lower in BO versus BC pre-lunch (*p* < 0.0005; d = 1.20), but not post-lunch, with TAUC being lower in BO versus BC across the condition (d = 0.91).

### 3.5. Exercise Responses

**Figure 4:**
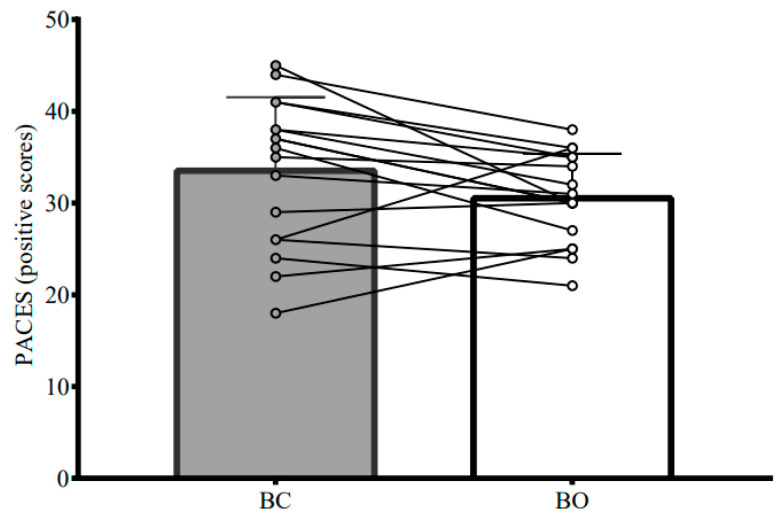
Physical Activity Enjoyment Scale responses (16-point scale) during cycling exercise performed 5 h after breakfast omission (BO) and breakfast consumption (BC). Data are presented as means and SD. Circles and lines represent individual data points. BC was the consumption of a standardised breakfast providing 0.06 g of carbohydrate per kcal of measured resting metabolic rate. BO was the consumption of an amount of water equivalent to the fluid content of the standardised breakfast provided in BC. A standardised lunch providing 0.08 g of carbohydrate per kcal of measured resting metabolic rate was provided 3 h after BO or BC. An incremental exercise protocol was performed 2 h after lunch. Statistical analyses were completed using linear mixed models.

Exercise responses during the incremental exercise protocol are shown in Table 4. Fatmax, peak fat oxidation rate, and ratings of perceived exertion were not significantly different between BO and BC. There was a strong tendency and small effect for lower physical activity enjoyment in BO versus BC (*p* = 0.055; d = 0.46), as shown in Figure 4.

### 3.6. Perceptions of Appetite and Mood

Perceived fullness (d = 1.89) and hunger (d = 1.35) were significantly lower and higher, respectively, in BO versus BC (*p* < 0.0005) with large effects; the significant condition by time interaction indicated significant differences at all pre-lunch time points excluding baseline (*p* ≤ 0003). Perceived energy (d = 1.12) and concentration (d = 0.49) levels were significantly lower in BO versus BC (*p* < 0.0005), whilst perceived tiredness was higher in BO versus BC (*p* = 0.001; d = 0.28). There was a significant condition by time interaction for concentration (*p* = 0.037), although post hoc analyses only revealed a significant difference at 2 h post-lunch. Calmness and tension did not differ significantly between the conditions (*p* ≥ 0.414).

## 4. Discussion

This study is the first to report higher post-lunch glycaemia and insulinaemia in response to breakfast omission versus breakfast consumption among adolescent girls who habitually consume breakfast, indicating a ‘second meal effect’. Moreover, the large magnitude of these post-lunch differences meant that glycaemia and insulinaemia were higher across the 5 h spanning pre- and post-lunch when breakfast was omitted versus consumed. This contributed to a higher MLI with breakfast omission, despite the lower TAG response. Carbohydrate oxidation and fat oxidation were lower and higher, respectively, with breakfast omission versus consumption; the effect for IAUC was reversed after lunch. Although Fatmax and peak fat oxidation 2 h after lunch were unaffected, omitting breakfast appeared to reduce exercise enjoyment.

The higher post-lunch glycaemia and insulinaemia in response to breakfast omission versus consumption suggests that adolescent girls respond similarly to adults [16,17,18], despite important metabolic changes that occur during the child–adult transition [6,26,27,28]. Due to pubertal insulin resistance affecting even healthy adolescents, eating carbohydrates earlier in the day (via breakfast) when insulin sensitivity and glucose tolerance are highest may be particularly important for this population, placing less demand on the pancreatic β-cells [24,25]. Even with their potentially lower capacity to store and use endogenous carbohydrate until mid- to late puberty [26,27], the girls in this study benefited from post-lunch reductions in glycaemia with breakfast consumption, where enhanced muscle glycogen storage is a key mechanism among adults [18]. The similar results with adult data may be because the majority of our sample self-reported as Tanner stage 4 where endogenous carbohydrate oxidation is comparable to adults among boys, although data among girls is limited [26,27,28]. Thus, our results may not apply to adolescents during the earlier stages of puberty. Increased pre-lunch non-esterified fatty acid concentrations appear to contribute to reduced glucose conversion into muscle glycogen after breakfast omission versus consumption [18]. Congruent with this finding, pre-lunch fat oxidation was higher with breakfast omission, indicating greater non-esterified fatty acid availability. The lower carbohydrate oxidation, as well as total resting energy expenditure, may have contributed further to the higher plasma glucose concentrations post-lunch with breakfast omission. Although not assessed in the present study, a faster rate of gastric emptying and thus glucose entry into the blood with breakfast omission may have been another influential mechanism for the second-meal effect reported here [29]. These metabolic responses did not translate to differences in Fatmax and peak fat oxidation, potentially because exercise was performed 2 h after lunch to coincide with the after-school period [62] and prevent interference with post-lunch responses. Indeed, carbohydrate consumed 45 min [73] and 1 and 3 h before exercise [74] reduced Fatmax and maximal fat oxidation in adults when no additional meals were consumed.

Regarding consistency, all but one of the girls in our study exhibited higher post-lunch glycaemia and insulinaemia and higher peak concentrations when they omitted breakfast. This, coupled with the small glucose response to the low GI high-fibre breakfast, meant that glycaemia was higher across the 5 h experimental period, despite the girls consuming less carbohydrate with breakfast omission. If repeated habitually, a series of higher overall and peak glycaemic responses induced by breakfast omission could contribute to cardiometabolic disease among adolescent girls [1]. This increased cardiometabolic disease risk is further supported by the higher MLI with breakfast omission, which reflects the combined glucose and TAG response rather than glucose alone. Yet, past data in mixed samples of adolescents and adults has not observed such clear differences in response to breakfast omission versus consumption, perhaps due to possible interactions with breakfast characteristics and habitual breakfast patterns [31,32]. For instance, three days of skipping breakfast or consuming a normal- or high-protein breakfast did not affect post-lunch glycaemia and insulinaemia assessed on day 4 among habitual breakfast skipping females (mean age 19 years) classified as overweight/obese [31]. This may be because the 350 kcal and 38–59 g carbohydrate consumed at breakfast was insufficient to suppress plasma non-esterified fatty acids for a subsequent glycaemic effect [51,75]. For comparison, the breakfast in our study provided ≈588 kcal with ≈94 g of carbohydrate and the breakfasts in past studies with adults provided ≈469–850 kcal with ≈82–100 g of carbohydrates [16,17,18]. Although previous work with mixed samples of adolescents and adults did not state the GI of the breakfasts [31,32], unlike our study, the foods included were also generally not low in GI [49] to help contribute to favourable glycaemic effects [52]. Our large and consistent effects across the sample may have also been due to the inclusion of apparently healthy habitual breakfast consumers. Indeed, blunted metabolic responses to breakfast manipulations were reported when comparing habitual breakfast skippers to consumers among overweight women [34], and the young females with overweight and obesity in past work [31,32] may exhibit metabolic flexibility, leading to blunted responses [76]. Taken together, these findings indicate particularly harmful effects of breakfast omission for girls who are accustomed to eating breakfast.

Reduced carbohydrate availability with breakfast omission may have contributed to the strong tendency for lower afternoon exercise enjoyment (i.e., 2 h post-lunch), as well as the lower energy levels and higher tiredness reported across the day when compared with breakfast consumption. Breakfast omission also increased hunger and decreased fullness pre-lunch, supporting past research among adolescent girls [39,77]. As enjoyment is a fundamental intrinsic motivational factor for physical activity engagement among adolescent girls [78,79], the potential for breakfast to improve physical activity through this pathway warrants investigation. Indeed, consuming breakfast more frequently may prompt higher free-living physical activity among adolescents [9,14], notwithstanding this finding is not entirely consistent [8]. Further, the lower perceived concentration with breakfast omission could have implications for cognitive function [80]. The acute ‘benefits’ of consuming breakfast may thus extend beyond cardiometabolic health to physical activity and academic performance.

The acute, controlled nature of this study was a strength due to the minimal influence of confounders, but did not permit an understanding of chronic and/or free-living effects. Our sample of healthy adolescent girls who habitually consumed breakfast is relevant for cardiometabolic disease prevention, which is more effective than a cure [81,82]. Yet, the findings may lack generalisability to girls who habitually skip breakfast or are at high risk of cardiometabolic disease. Further, chronotype, circadian rhythms, physical activity habits, and breakfast meal characteristics could influence the findings, which warrant examination. Although attempts were made to minimise the possible confounding influence of menstrual cycle phase by aligning the experimental conditions to the early follicular phase, this was not possible due to sporadic menstrual cycles and logistics of testing in pairs. That said, differences in insulin sensitivity and exercise metabolism with menstrual cycle phase have not consistently been reported and are based on adult rather than adolescent data [43,44]. 

To conclude, we report for the first time that breakfast omission increases subsequent glycaemia and insulinaemia when compared with breakfast consumption among adolescent girls who habitually consume breakfast. Omitting breakfast may also reduce afternoon exercise enjoyment, with Fatmax and exercise peak fat oxidation being unaffected. These findings suggest effective strategies are required to promote sustained breakfast consumption among adolescent girls for cardiometabolic disease prevention.

## Figures and Tables

**Table 1:** Characteristics of adolescent girls who participated in randomised crossover trial comparing breakfast omission (BO) with breakfast consumption (BC) ^1^.

	Value ± Variability
Age (years)	13.2 ± 0.7
Stature (m)	1.56 ± 0.06
Body mass (kg)	51.2 ± 13.9
Body fat %	27.6 ± 7.0
Waist circumference (cm)	66.8 ± 9.1
Body mass index (kg∙m^−2^)	20.9 ± 4.9
Breast development (stage) ^2^	4 (1)
Pubic hair (stage) ^2^	4 (1)
Resting metabolic rate (kcal/day)	1566 ± 244

^1^ Values are mean ± SDs or medians (IQRs), *n* = 17. ^2^ Five stages of breast and pubic hair development described by Tanner [37].

**Table 2:** Absolute energy and macronutrient intakes of breakfast and lunch meals.

	Breakfast	Lunch
Energy (kcal)	588 ± 92	768 ± 120
Carbohydrate (g)	93.9 ± 14.6	125.3 ± 19.5
Fat (g)	8.9 ± 1.4	23.0 ± 3.6
Protein (g)	21.0 ± 3.3	11.3 ± 1.8
Fibre (g)	20.7 ± 3.2	4.6 ± 0.7

Values are mean ± SD, *n* = 17. Breakfast and lunch were provided to the participants in quantities containing 0.06 g and 0.08 g of carbohydrate per kcal of individual measured resting metabolic rate, respectively.

**Table 3:** Area under the curve analyses of cardiometabolic risk marker responses to breakfast omission (BO) versus breakfast consumption (BC) among adolescent girls classified as habitual breakfast consumers ^1^.

	Pre-Lunch (3 h)		Post-Lunch (2 h)		*p*	
	BC	BO	BC	BO	Condition Main Effect	Condition by Time Interaction
Glucose IAUC (mmol·L^−1^)	**81** **(−1, 163)**	**−55** **(−137, 27)**	**318** **(235, 400)**	**559** **(477, 641)**	0.210	<0.0005
Glucose TAUC (mmol·L^−1^)	**999** **(919, 1080)**	**879** **(799, 960)**	**1163** **(1083, 1244)**	**1347** **(1266, 1427)**	0.395	0.001
Insulin IAUC (pmol·L^−1^)	**4203** **(2399, 6007)**	**−284** **(−2085, 1516)**	**7907** **(6103, 9711)**	**14,772** **(12,972, 16,573)**	0.145	<0.0005
Insulin TAUC (pmol·L^−1^)	**5655** **(3830, 7479)**	**1197** **(−624, 3017)**	**9889** **(8064, 11,713)**	**15,621** **(13,800, 17,441)**	0.413	<0.0005
TAG IAUC (mmol·L^−1^)	5.2 (−4.8, 15.2)	−0.5 (−10.5, 9.5)	1.3 (−8.7, 11.3)	−13.0 (−23.0, −3.0)	0.070	0.430
TAG TAUC (mmol·L^−1^)	157 (145, 169)	151 (138, 163)	143 (130, 155)	114 (102, 127)	0.004	0.060
Metabolic load index IAUC (mmol·L^−1^)	**89** **(−2, 180)**	**−53** **(−144, 38)**	**323** **(232, 414)**	**568** **(476, 659)**	0.264	<0.0005
Metabolic load index TAUC (mmol·L^−1^)	**1164** **(1076, 1251)**	**1036** **(949, 1123)**	**1317** **(1229, 1404)**	**1485** **(1398, 1572)**	0.608	0.001
Fat oxidation IAUC (mg)	**−3966** **(−5049, −2883)**	**1223** **(140, 2307)**	**−853** **(−1936, 230)**	**−3083** **(−4167, −1999)**	0.016	<0.0005
Fat oxidation TAUC (mg)	**5561** **(4418, 6704)**	**10,209** **(9064, 11,355)**	**2895** **(1752, 4038)**	**5014** **(3868, 6160)**	<0.0005	0.015
Carbohydrate oxidation IAUC (mg)	**19,293** **(16,177, 22,409)**	**−299** **(−3418, 2820)**	**6774** **(3658, 9890)**	**11,179** **(8060, 14,297)**	<0.0005	<0.0005
Carbohydrate oxidation TAUC (mg)	**50,129** **(47,062, 53,196)**	**31,300** **(28,230, 34,369)**	**39,864** **(36,798, 42,931)**	**31,135** **(28,065, 34,205)**	<0.0005	<0.0005
Energy expenditure IAUC (kcal)	**41.6** **(27.6, 55.5)**	**9.8** **(−4.2, 23.7)**	3.3 (−10.7, 17.3)	1.9 (−12.0, 15.9)	0.003	0.006
Energy expenditure TAUC (kcal)	251 (235, 266)	217 (202, 233)	183 (167, 198)	168 (153, 184)	<0.0005	0.067

^1^ Values are marginal mean (95% confidence intervals); *n* = 15 for plasma glucose; *n* = 16 for insulin; *n* = 13 for TAG; *n* = 17 for substrate oxidation and energy expenditure. BC was the consumption of a standardised breakfast providing 0.06 g of carbohydrate per kcal of measured resting metabolic rate. BO was the consumption of an amount of water equivalent to the fluid content of the standardised breakfast provided in BC. A standardised lunch providing 0.08 g of carbohydrate per kcal of measured resting metabolic rate was provided 3 h after BO or BC. Pre-lunch values are over 3 h (i.e., between BO or BC and lunch) and post-lunch values are over 2 h (i.e., the 2 h period after lunch). Bold text indicates significant between-condition difference within time segment. Statistical analyses were completed using linear mixed models with condition and time included as fixed factors. IAUC = incremental area under the curve; TAUC = total area under the curve; TAG = triacylglycerol.

**Table 4:** Exercise responses to breakfast omission (BO) versus breakfast consumption (BC) among adolescent girls classified as habitual breakfast consumers ^1^.

	BC	BO	*p*
Fatmax (heart rate, beats·min^−1^)	125 (122, 137)	124 (112, 137)	0.944
Peak fat oxidation rate (mg·min^−1^)	49 (36, 62)	59 (46, 72)	0.095
Perceived exercise exertion	4 (3, 4)	4 (3, 5)	0.306
Physical activity enjoyment	**34** **(30, 37)**	**31** **(27, 34)**	**0.055**

^1^ Values are marginal mean (95% confidence intervals); *n* = 17. BC was the consumption of a standardised breakfast providing 0.06 g of carbohydrate per kcal of measured resting metabolic rate. BO was the consumption of an amount of water equivalent to the fluid content of the standardised breakfast provided in BC. A standardised lunch providing 0.08 g of carbohydrate per kcal of measured resting metabolic rate was provided 3 h after BO or BC. An incremental exercise protocol was performed 2 h after lunch. Bold text indicates a difference that is significant or approaching significance between BO and BC. Statistical analyses were completed using linear mixed models with condition included as a fixed factor. Perceived exertion was assessed using the 10-point OMNI [67]. Physical activity enjoyment was assessed using the 16-point Physical Activity Enjoyment [68].

## Data Availability

Data described in the manuscript will be made available upon request, pending application and approval.
